# Pelvic Hematocele and Sub Serous Fibroid

**Published:** 1873-12

**Authors:** T. G. Thomas


Pelvic Hæmatocele and Sub-serous Fibroid—*By Prof. T. G. Thomas.*—Gentlemen: The patient before us says that four months ago she complained of severe pain in abdomen, with pains over the whole body. She came under observation at that time, though she had been seen before, and she was found to have a sub-serous fibroid tumor. What she does not say anything about, and what she did not know of, was the development of a large tumor in the abdomen as large as my head. Now it was found, when this tumor made its appearance, that both uterus and uterine tumor disappeared in the mass. During the month of last July, it was beginning to be absorbed, and now it is gone, but the fibroid has again made its appearance. The important question is, what was it? It was a case of pelvic hæmatocele which has passed through the ordinary course of absorption. The patient has been sterile, as is usually the case with patients having a uterine fibroid. The tumor, dragging on the organ, changes, in all probability, the canal of the cervix. As regards treatment, I have no confidence in the world in the beneficial use of drugs to reduce the growth. But there are those who think that the bromide of ammonium or potassium will cause absorption. Now if such an observer had met this case of hæmatocele, and not made out its character, he would have sworn to his grandchildren that he reduced a tumor from the size of his head down to that of a goose egg.

